# Patterns of Smoking Prevalence among the Elderly in Europe

**DOI:** 10.3390/ijerph10094418

**Published:** 2013-09-17

**Authors:** Alessandra Lugo, Carlo La Vecchia, Stefania Boccia, Bojana Murisic, Silvano Gallus

**Affiliations:** 1 Department of Epidemiology, IRCCS – Istituto di Ricerche Farmacologiche “Mario Negri”, 20156 Milan, Italy; E-Mails: alessandra.lugo@marionegri.it (A.L.); carlo.lavecchia@marionegri.it (C.L.V.); bojana.murisic@marionegri.it (B.M.); silvano.gallus@marionegri.it (S.G.); 2 Department of Clinical Sciences and Community Health, Università degli Studi di Milano, 20133 Milan, Italy; 3 Section of Hygiene, Institute of Public Health, Università Cattolica del Sacro Cuore, 00168 Rome, Italy; 4 IRCCS San Raffaele Pisana,00163 Rome, Italy

**Keywords:** smoking prevalence, vulnerable population, elderly, cross-sectional study

## Abstract

Scant information is available on determinants of smoking prevalence in the vulnerable population of the elderly, particularly in Europe. Therefore, we analyzed smoking patterns among older adults (≥65 years old), using data from a representative survey based on 3,071 elderly, conducted in 17 European countries in 2010, within the Pricing Policies And Control of Tobacco in Europe (PPACTE) project. Overall smoking prevalence in 17 European countries was 11.5% (15.3% in men and 8.6% in women). An inverse relation with level of education was observed among men, while no specific pattern was evident among women. Smoking prevalence was highest in eastern/central Europe for men (20.3%) and northern Europe for women (13.1%). In both sexes combined, smokers were more frequent in countries with low implementation of tobacco control activities (14.9%). Anti-tobacco campaigns and smoking cessation interventions specifically targeted to the elderly are urgently needed in Europe.

## 1. Introduction

The association between cigarette smoking and the risk of various diseases and shorter life expectancy has been widely demonstrated [1,2,3]. In older age, substantially higher mortality from cancers, respiratory and cardiovascular illnesses and other chronic conditions has been observed in smokers compared to non-smokers [3,4]. In a recent meta-analysis based on 17 cohort studies on the elderly, smokers had an 83% increased all causes mortality compared to never smokers. Former smokers had a substantially lower excess risk (34%), which decreased with increasing time since stopping [5]. The cohort of British doctors, based on 36,000 males followed for 50 years, quantified the beneficial effects of smoking cessation on survival at different ages: even a 60 year old cigarette smoker could increase his life expectancy by 30% by stopping smoking [1]. Thus, smoking cessation, even at advanced age, has a major positive impact on human health [6,7,8].

Several reasons exist why smoking cessation is difficult among older smokers, including longer duration of smoking habit, higher number of cigarettes smoked daily, lower intention and fewer attempts to quit, and higher rate of hardcore smokers among elderly smokers [9,10,11]. Moreover, reasons to quit smoking among older and younger smokers may be different [12,13,14,15]. In order to find the best approach to support smoking cessation in the elderly, it is important to study smoking behaviours and patterns in this vulnerable population [7].

Only a few studies were specifically designed to analyze the socio-demographic characteristics influencing smoking behavior in the elderly [14,15,16,17,18,19]. In Europe, a large volume of literature on the monitoring of smoking prevalence focuses on adolescents and young adults [20,21,22,23], but little attention has been paid to the elderly [15]. This is particularly worrisome, given that over the last few decades the burden of smoking-related diseases in the elderly has become greater and greater.

The aim of this study is to describe smoking patterns among the elderly in 17 European countries from a survey conducted in 2010 within the Pricing Policies And Control of Tobacco in Europe (PPACTE) project [24,25,26].

## 2. Materials and Methods

### 2.1. European Survey

Within the PPACTE project, a face-to-face multicounty cross-sectional survey on smoking was conducted between January and July 2010 in 18 selected European countries (Albania, Austria, Bulgaria, Czech Republic, Croatia, England, Finland, France, Greece, Hungary, Ireland, Italy, Latvia, Poland, Portugal, Romania, Spain and Sweden). The survey was coordinated by Doxa, the Italian branch of the Worldwide Independent Network/Gallup International Association (WIN/GIA), and its European partners [24,25,26]. In each country, we enrolled a sample of around 1,000 individuals representative of the country-specific population aged 15 years or older in terms of age, sex, geographic area and socio-economic characteristics. We defined elderly as people aged 65 years or older. Since our sample in Greece included participants younger than 65, only, we excluded Greece from our analysis. Therefore, our study is based on 17 European countries, including a total of 3,071 participants (1,430 men and 1,641 women). 

Sampling methodologies were different across various countries. In several countries (Albania, Croatia, Hungary, Italy, Poland and Romania) a multi-stage methodology was used. In the first stage, the primary unit of selection was a geographic area or voting centre. In the second stage, households or municipalities were selected. In the last stage, respondents were chosen randomly, in order to be representative of the population in terms of sex, age, geographic area and socio-economic characteristics (working status, occupation and income). For other countries (Austria, England, Finland, France and Ireland) we used a quota method for the selection of the entire sample, stratifying the population according to selected variables including age, sex, and alternatively geographic area and/or occupation, in order to obtain a representative sample of the country population. For other countries, we used other sampling methodologies, including a stratified random method for Bulgaria, the Czech Republic and Latvia. Given the different sampling methodologies used, various surveys had heterogeneous response rates. Full details of the survey methodology and response rates are reported elsewhere [25,26]. Most of the countries used statistical weights in order to assure the representativeness of the sample. 

Data were collected by trained interviewers using a standardized questionnaire. Besides information on socio-demographic characteristics, information on smoking status was collected. Current smokers were defined as those who had smoked 100 or more cigarettes in their lifetime and were smokers when the survey took place. Ex-smokers were those who had quit before the survey took place, since current smokers were asked to show their latest cigarette pack. Ever smokers were asked about their age at starting, the number of cigarettes smoked per day and the time to first cigarette after waking up. Based on the latter two items, for current smokers we computed the Heavy Smoking Index (HSI), an index of tobacco dependence, with an overall score ranging from 0 to 6 [27]. Current smokers were asked whether they had an intention to quit within the following 6 months. Ex-smokers were asked to report the age at quitting and the most important reason that led them to quit. Reasons to quit were categorized as follows: (a) illness (any medical condition); (b) physician’s advice; (c) knowledge of the harmful effects of smoking; (d) smoke-free legislation; (e) pregnancy/birth of a child; (f) economic reason (cigarettes too expensive); (g) pressure to quit by partner/relatives; (h) employer reluctance to hire smokers; (i) other reasons. 

Education was grouped into three categories (low/intermediate/high) by Doxa and its European partners, according to the country-specific school system. With reference to geographic area, countries were categorized as northern, western, southern and central/eastern Europe. For the European Union (EU) Member States (MS), the Tobacco Control Scale (TCS) score, updated to 2010 [28], was used to distinguish countries with limited implementation of tobacco control policies (TCS < 45. 45 being the median value for the countries considered) from those with a better implementation (TCS ≥ 45). Albania and Croatia, not EU MS, were excluded from the TCS stratification analysis. 

The study protocol was approved by the Institutional Review Board of the Istituto di Ricerche Farmacologiche “Mario Negri”. The procedures for recruitment of subjects, informed consent, data collection, storage and protection (based on anonymous identification code) are in accordance with the current country specific legislation. This was ratified and signed by Doxa and each of its European partners.

### 2.2. Statistical Methods

To estimate smoking prevalence and other outcomes for the overall sample of the European survey data, we further applied a weighting factor, in addition to country-specific statistical weight, with each country contributing in proportion to its population aged 15 years or older. The country-specific population aged ≥15 years was obtained by Eurostat [29]. For strata of education, we standardized smoking prevalence estimates by two categories of age (65–74 and ≥75 years old), using the direct method on the total sample of men and women 

## 3. Results

**Figure 1:**
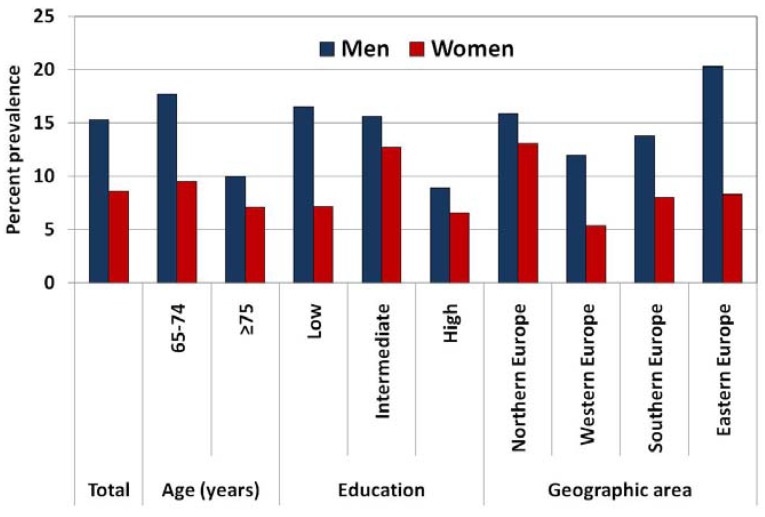
Smoking prevalence (%) among the elderly (≥65 years) in 17 European countries, overall and according to age, level of education and geographic area.

Table 1 and Figure 1 show the prevalence of current and ex-smokers among 3,071 European individuals aged ≥65 years. Overall, 11.5% were current smokers (15.3% of men and 8.6% of women), 23.5% ex-smokers (33.9% of men and 15.2% of women) and 65.0% never smokers (50.9% of men and 76.2% of women). Smoking prevalence in the elderly was lowest in Austria (6.6%), Sweden (7.4%) and France (8.3%) and highest in Ireland (21.9%), Hungary (25.7%) and Albania (30.3%). Smoking prevalence in men ranged from 9.0% (Sweden) to 40.5% (Albania), and in women from 2.3% (Latvia) to 28.1% (Ireland). 

Smoking prevalence was significantly more frequent among Europeans aged 65–74 year (13.4%) than those aged ≥75 years (8.2%) overall and in men, but not in women. In men, age-adjusted smoking prevalence was highest in elderly with low level of education (16.5%) and lowest in those with a high level of education (8.9%; *p* for trend = 0.015). 

**Table 1:** Percent (%) prevalence *, and corresponding 95% confidence interval (CI), of current and ex-smokers among 3,071 Europeans aged ≥65years (1,430 men and 1,641 women), according to selected characteristics, overall and by sex. PPACTE, 2010.

	N	Current smokers (%; 95% CI)			Ex-smokers (%; 95% CI)		
		Total	Men	Women	Total	Men	Women
**Total**	3,071	11.5 (10.4–12.6)	15.3 (13.4–17.2)	8.6 (7.2–10.0)	23.5 (22.0–25.0)	33.9 (31.4–36.4)	15.2 (13.5–16.9)
Age							
65–74	2,029	13.4 (11.9–14.9)	17.7 (15.3–20.1)	9.5 (7.7–11.3)	23.0 (21.2–24.8)	31.1 (28.2–34.0)	15.8 (13.6–18.0)
≥75	1,042	8.2 (6.5–9.9)	10.0 (7.3–12.7)	7.1 (5.0–9.2)	24.4 (21.8–27.0)	39.9 (35.5–44.3)	7.1 (5.0–9.2)
Education^							
Low	1,704	11.6 (10.1–13.1)	16.5 (13.9–19.1)	7.2 (5.5–8.9)	21.1 (19.2–23.0)	31.7 (28.5–34.9)	11.6 (9.5–13.7)
Intermediate	954	14.0 (11.8–16.2)	15.6 (12.1–19.1)	12.7 (9.9–15.5)	30.0 (27.1–32.9)	39.3 (34.6–44.0)	22.1 (18.6–25.6)
High	412	7.7 (5.1–10.3)	8.9 (5.0–12.8)	6.6 (3.2–10.0)	30.0 (25.6–34.4)	34.5 (28.0–41.0)	25.4 (19.4–31.4)
Geographic area							
Northern Europe	844	14.4 (12.0–16.8)	15.9 (12.3–19.5)	13.1 (10.0–16.2)	41.6 (38.3–44.9)	52.0 (47.1–56.9)	32.9 (28.6–37.2)
Western Europe	380	8.2 (5.4–11.0)	12.0 (7.1–16.9)	5.4 (2.4–8.4)	27.2 (22.7–31.7)	47.2 (39.6–54.8)	12.8 (8.3–17.3)
Southern Europe	577	10.6 (8.1–13.1)	13.8 (9.7–17.9)	8.0 (5.0–11.0)	14.8 (11.9–17.7)	21.9 (16.9–26.9)	9.1 (5.9–12.3)
Eastern and central Europe	1,270	13.8 (11.9–15.7)	20.3 (17.1–23.5)	8.3 (6.2–10.4)	16.6 (14.6–18.6)	23.8 (20.4–27.2)	10.6 (8.3–12.9)
Tobacco Control Scale (TCS) #							
<45	1,103	14.9 (12.8–17.0)	20.6 (17.0–24.2)	10.3 (7.9–12.7)	19.1 (16.8–21.4)	24.3 (20.5–28.1)	14.9 (12.1–17.7)
≥45	1,712	10.8 (9.3–12.3)	14.0 (11.6–16.4)	8.2 (6.4–10.0)	24.7 (22.7–26.7)	36.2 (32.9–39.5)	15.6 (13.2–18.0)

* Prevalence estimates were computed weighting each country in proportion to the country specific population aged 15 years or older; ^ The sum does not add up to the total because of some missing values. According to level of education smoking prevalence estimates have been standardized by age; Classification of countries. Northern Europe: FI, IE, SE, UK; Western Europe: AT, FR; Southern Europe: ES, GR, IT, PT; Eastern and Central Europe: AL, BG, CZ, HR, HU, LV, PL, RO. TCS < 45: AT, BG, CZ, HU, LV, PL, PT; TCS ≥ 45: ES, FI, FR, IE, IT, RO, SE, UK; # Albania and Croatia were excluded.

In women age-adjusted smoking prevalence was highest in those with an intermediate level of education (12.7%) without any linear trend with education (*p* for trend = 0.178). In both sexes, ex-smokers were less frequent among lowest educated subjects. 

Smoking prevalence was 14.4% in northern European countries, 8.2% in western, 10.6% in southern and 13.8% in eastern/central Europe. In men smoking prevalence was highest in eastern and central European countries (20.3%), while in women in northern European ones (13.1%). Current smokers were more frequent in countries with TCS < 45 (14.9%) as compared to countries with TCS ≥ 45 (10.8%; *p* = 0.004), whereas ex-smokers were more frequent in countries with TCS ≥ 45 (24.7%) compared to countries with TCS < 45 (19.1%; *p* = 0.003). The same patterns were observed in men, while no significant difference according to country-specific characteristics was observed in female smoking prevalence. 

For ever smokers, average age at starting was 19.6 years (SD 7.3) overall, 18.0 years (SD 4.8) among men and 22.3 years (SD 9.9) among women. The corresponding estimates for current smokers were 19.6 years (SD 8.1), 17.7 (SD 5.5) and 22.7 (SD 10.7), respectively.

Among current smokers, 36.2% (38.8% of men and 32.3% of women) had an intention to quit smoking within the following 6 months. 

The mean number of cigarettes per day for current smokers was 15.8 (SD 12.1) overall, 16.7 (SD 13.5) in men and 14.3 (SD 8.9) in women. Overall, 20.5% of current smokers lighted their first cigarette within 5 min after waking up, 34.5% within 6 to 30, 16.3% within 31 to 60 min and 28.8% over 1 h after waking up. Among current smokers 76.9% had an HSI score <4 (relatively low dependence; 77.5% in men and 76.2% in women) and 23.1% of current smokers had a HSI score ≥4 (high dependence; 22.5% in men and 23.8% in women; *p* = 0.634).

Table 2 shows the percent distribution of ex-smokers, aged 65 years or over at the time when the study took place, according to the most important reason that led them to quit smoking. Overall, illness was the most frequently reported reason for smoking cessation (39.8%), followed by knowledge of harmful effects of smoking (19.5%), physician’s advice (9.6%), economic reasons (5.3%), pressure by partner/relatives (4.0%), pregnancy or birth of a child (2.4%), smoke-free legislation (0.3%) and employer reluctance to hire smokers (0.1%). Ex-smokers reporting medical conditions as the main reason to quit were more frequently men (42.1%), aged 65–74 years (40.6%), less educated (45.0%), living in southern European countries (52.4%) and in countries with a high implementation of tobacco control activities (41.5%).

## 4. Discussion

Using a large representative survey conducted in 17 European countries, we found that the overall smoking prevalence among adults aged ≥65 years was 12% (15% among men and 9% among women). Considering only individuals aged ≥75 years, the corresponding prevalence was 8% overall, 10% in men and 7% in women. Our estimates are in broad agreement with those provided by the Survey of Health, Ageing and Retirement in Europe (SHARE), a cross-sectional study based on 26,743 adults aged ≥50 years, conducted in 11 western, central and southern European countries in 2004–2005.In that survey, smoking prevalence for adults aged 70–79 years was 14% in men and 8% in women. The corresponding estimates for adults aged ≥80 years were 10% and 4%, respectively [30]. 

**Table 2:** Percent distribution * of European ex-smokers aged ≥65 years, according to the most important reason that led them to quit smoking, overall and in strata of selected characteristics. PPACTE, 2010.

		N ex smokers aged ≥65	Reason to quit (%)								
			Illness (any medical condition)	Knowledge of the harmful effects of smoking	Physician’s advice	Economic reasons (cigarettes too expensive)	Pressure to quit by partner/ relatives	Pregnancy/birth of a child	Smoke-free legislation	Employer reluctance to hire smokers	Other reasons
**Total**		693	39.8	19.5	9.6	5.3	4.0	2.4	0.3	0.1	19.1
Sex											
	Men	465	42.1	19.7	10.2	4.2	2.7	1.6	0.4	0.1	19.0
	Women	228	35.7	19.2	8.5	7.5	6.3	3.7	0.0	0.0	19.3
Age (years)											
	65–74	453	40.6	23.0	9.1	5.3	2.9	1.8	0.4	0.1	16.8
	≥75	240	38.3	13.6	10.4	5.4	5.8	3.9	0.0	0.2	23.0
Education^											
	Low	339	45.0	15.8	10.6	4.8	2.6	3.0	0.0	0.1	18.1
	Intermediate	236	33.3	21.8	9.9	7.4	5.6	1.5	0.9	0.0	19.6
	High	117	31.7	29.3	4.2	2.9	7.0	1.6	0.1	0.3	22.9
Geographic area											
	Northern Europe	291	32.3	20.9	8.5	8.9	4.0	1.5	0.0	0.1	23.8
	Western Europe	99	39.6	12.8	7.6	1.6	0.4	4.3	0.0	0.0	33.7
	Southern Europe	93	52.4	27.1	7.4	1.1	8.8	1.9	1.1	0.3	0.0
	Eastern and central Europe	210	38.9	14.3	19.3	9.2	2.3	2.3	0.0	0.0	13.6
Tobacco Control Scale (TCS) #											
	<45	212	28.7	14.6	27.9	9.4	5.1	6.1	0.0	0.4	7.9
	≥45	446	41.5	20.4	6.5	4.7	3.9	1.8	0.3	0.0	20.9

* Prevalence estimates were computed weighting each country in proportion to the country specific population aged 15 years or over; ^ The sum does not add up to the total because of some missing values; ° Classification of countries. Northern Europe: FI, IE, SE, UK; Western Europe: AT, FR; Southern Europe: ES, GR, IT, PT; Eastern and central Europe: AL, BG, CZ, HR, HU, LV, PL, RO. TCS<45: AT, BG, CZ, HU, LV, PL, PT; TCS ≥45: ES, FI, FR, IE, IT, RO, SE, UK; # Albania and Croatia were excluded.

According to the model of the tobacco epidemic proposed by Lopez and colleagues in 1994, early stages of the tobacco epidemic are characterized by a direct relation between socio-economic status and smoking prevalence, while the late stages are characterized by an inverse relation, given that individuals with a high socio-economic status respond more favourably to health promotion campaigns [31]. In women, the switch from direct to inverse relation occurs later than among men. This is due to the fact that smoking prevalence in women typically lags behind that of men by a few decades [31]. A US study using repeated cross-sectional data from seven National Health Interview Surveys (NHIS) conducted between 1965 and 1994 showed that among the elderly (≥65) the relation between level of education and smoking prevalence was direct in the 1970s, null in the 1980s and slightly inverse in the 1990s. In the same dataset, younger adults (18–64 years old) showed an inverse relation already in the 1970s, which became stronger in 1980s and strongest in 1990s [7]. Accordingly, a study based on a large representative survey, the US National Surveys on Drug Use and Health (NSDUH), conducted in 2008 and 2009, confirmed a weaker inverse relation, in both sexes combined, between education and smoking prevalence among the elderly compared with middle-aged adults [32]. This suggests that older adults (*i.e*., earlier cohorts) are in an earlier stage of the tobacco epidemic, compared to younger adults. Our results on the relation between education and smoking in the European elderly are in broad agreement with this line of reasoning. Thus, whereas highest educated men had the lowest age-adjusted prevalence of smoking, in elderly women we did not observe a clear pattern. On the contrary, in the general population the level of education is inversely related to smoking prevalence, not only in men, but also in women [26]. Thus, although cessation rates are highest among more educated women at an advanced age [33], their smoking prevalence remains considerably high.

We found substantial differences in various European countries in terms of smoking prevalence, ranging between 7% in Austria and 26% in Hungary. Male-to-female smoking prevalence ratio and current-to-ex smoking prevalence ratio may be taken as marks of the evolution of the tobacco epidemic, with the lowest values of both ratios usually coming at advanced stages of the tobacco epidemic [26,31,34,35]. In our population, large differences across countries were observed in terms of male-to-female smoking prevalence ratio (2.44 in eastern and central Europe, 2.22 in western, 1.72 in southern and 1.21 in northern Europe) and in terms of current-to-ex smoking prevalence ratio (0.83 in eastern and central Europe, 0.71 in southern, 0.35 in northern and 0.30 in western Europe). The heterogeneities in terms of both ratios that we observed across countries, clearly point to the different stages of the tobacco epidemic.

In agreement with findings for the general population in Europe [26,36,37,38], smoking prevalence in the elderly was lower in countries with a high tobacco control activity, indicating that the implementation of population-level interventions and anti-tobacco programs, is effective in increasing smoking cessation. Such a relationship has been observed in men but not in women, suggesting that tobacco control programs do not pay enough attention to (elderly) women or that they might not be equally effective as in men [26].

Among older smokers, 36% had an intention to quit within the next 6 months, with no evident difference compared to the overall adult population [25]. This is in apparent contrast with a study from Germany [39] and a survey conducted in the UK, the USA, Canada and Australia [40], which show that in elderly the intention to quit occurs less frequently than in younger adults. 

Although older smokers are more frequently hardcore smokers than younger ones [10], studies from Italy [41,42], Germany [43] and Brazil [44] found that elderly smokers have a lower smoking dependence than middle-aged ones. In our elderly population, 23% of current smokers were highly nicotine dependent (using an HSI score ≥4, according to previous publications [27,45,46]). This estimate was somewhat higher than those found in national representative surveys among the general adult population in Italy in 2002–2003 (18%) [42] and Spain in 2004–2005 (15%) [27]. Elderly smokers may however include a large proportion of subjects with psychological besides physical addiction.

Approximately 40% of European ex-smokers aged ≥65 years reported medical problems, and presumably smoking related conditions, as their main reason for quitting smoking. In agreement with a study on the general Italian adult population, this proportion was highest among men and less educated subjects [13]. Our results are consistent with a large body of evidence, showing that the most common reason for quitting is illness [13,47] and that a diagnosis of smoking-related condition is a strong determinant of smoking cessation in the elderly (“ill quitter” effect) [8,47,48,49,50,51]. “Ill quitters” are at a high risk of mortality or morbidity [13], since they quit too late, only after they have already had symptoms of a serious tobacco-related disease [13,47,51]. Ex-smokers from North and Western Europe, as well as countries with better implementation of tobacco control policies, more frequently reported “other reasons” as an important reason for quitting. Those “other reasons” may include health campaigns, higher social pressure and the desire to give a good role model to their family members.

Elderly smokers should keep in mind the harmful effects of smoking and the benefit of smoking cessation, especially before occurrence of the first smoking related symptoms. In our population only less than 20% of ex-smokers reported “knowledge of the harmful effects of smoking” as their main reason to quit. Thus, awareness of the beneficial effects of smoking cessation at all ages, including older ages, possibly through new warning labels on tobacco packs, may have important implication from a public health perspective in this vulnerable population. 

Still, only 10% of European ex-smokers quit following an advice from their physicians. This is likely due to the limited efforts of physicians and other health care providers in advising smokers, and specifically older smokers [52], to quit [53]. Smokers are more likely to quit if advised and encouraged by the physicians and other health care professionals [54]. This is even more important for older adults, since they have more frequently contacts with physicians and are more responsive to their advice to quit [55]. This notwithstanding, the elderly tend less often to receive from their physicians an advice to quit [55,56,57].

Only 5% of ex-smokers stopped primarily because of the economic cost of tobacco, indicating that cigarette prices are not sufficiently high to efficiently contribute to smoking cessation in Europe [13].

The limitations of this study include those inherent to its cross-sectional design. In addition, the same sampling methodology was not used in all countries. Moreover, the number of elderly in individual countries was relatively small. Strengths of our survey include the representativeness of the population of 17 European countries, the questionnaire drafted by a group of tobacco control experts, the use of a standardized definition of smokers, and the use of a face-to-face interview. 

## 5. Conclusions

Several aspects, of our survey indicate that older adults are in an earlier, and therefore less favourable, stage of the tobacco epidemic compared to younger adults, reflecting the attitudes of the corresponding birth cohort. Anti-tobacco campaigns and smoking cessation interventions specifically targeted to the elderly are urgently needed in Europe as well as in other countries [10,17]. 
